# Using the maximum clustering heterogeneous set-proportion to select the maximum window size for the spatial scan statistic

**DOI:** 10.1038/s41598-020-61829-y

**Published:** 2020-03-17

**Authors:** Wei Wang, Tao Zhang, Fei Yin, Xiong Xiao, Shiqi Chen, Xingyu Zhang, Xiaosong Li, Yue Ma

**Affiliations:** 1 West China School of Public Health and West China Fourth hospital, Sichuan University, Chengdu, China; 2 Women and Children’s Health Management Department, Sichuan Provincial Hospital for Women and Children, Chengdu, China; 3 Department of Systems, Populations and Leadership, University of Michigan, School of Nursing, Ann Arbor, United States

**Keywords:** Diseases, Health care, Epidemiology

## Abstract

The spatial scan statistic has been widely used to detect spatial clusters that are of common interest in many health-related problems. However, in most situations, different scan parameters, especially the maximum window size (MWS), result in obtaining different detected clusters. Although performance measures can select an optimal scan parameter, most of them depend on historical prior or true cluster information, which is usually unavailable in practical datasets. Currently, the Gini coefficient and the maximum clustering set-proportion statistic (MCS-P) are used to select appropriate parameters without any prior information. However, the Gini coefficient may be unstable and select inappropriate parameters, especially in complex practical datasets, while the MCS-P may have unsatisfactory performance in spatial datasets with heterogeneous clusters. Based on the MCS-P, we proposed a new indicator, the maximum clustering heterogeneous set-proportion (MCHS-P). A simulation study of selecting the optimal MWS confirmed that in spatial datasets with heterogeneous clusters, the MWSs selected using the MCHS-P have much better performance than those selected using the MCS-P; moreover, higher heterogeneity led to a larger advantage of the MCHS-P, with up to 538% and 69.5% improvement in the Youden's index and misclassification in specific scenarios, respectively. Meanwhile, the MCHS-P maintains similar performance to that of the MCS-P in spatial datasets with homogeneous clusters. Furthermore, the MCHS-P has significant improvements over the Gini coefficient and the default 50% MWS, especially in datasets with clusters that are not far from each other. Two practical studies showed similar results to those obtained in the simulation study. In the case where there is no prior information about the true clusters or the heterogeneity between the clusters, the MCHS-P is recommended to select the MWS in order to accurately identify spatial clusters.

## Introduction

With the development of geographic information systems, the global positioning system and remote sensing, a large number of health-related datasets with geographic locations are being collected. Accurately identifying the spatial variability, such as the difference in disease incidence between different locations, plays an important role in the elucidation of the potential causes of illnesses, allocation of limited health resources, formulation of rational public health policies and exploration of the characteristics of health-related problems. Kulldorff’s spatial scan statistic (SSS)^1^ is one of the most commonly used methods^2–5^ for identifying such a difference, namely, the region that is significantly different from the other regions and is known as a cluster. However, even when employing the SSS for the same dataset, different scan parameters will usually result in different results with different accuracy. For instance, in Ribeiro’s simulation study^6^, different parameters led to highly different powers, sensitivity and positive predictive values in the same data, with the largest differences reaching 0.9404, 0.9369, and 0.825, respectively. Tango’s simulation study^7^ showed that the difference in the power between different parameters reached 0.596 (0.738 vs 0.142). In these scan parameters, the maximum spatial window size (MWS), commonly defined as a percentage of the maximum population in a single cluster, has very strong effect on the detected result^6,8^. The MWS is set to the default value of 50% in most studies and in some cases this leads to detecting an overly large region falsely covering regions between several small real clusters. Smaller MWSs are also selected for practical reasons, including limited resources for intervention^6^, special terrain^9^, spatial location discontinuity^10^, and other reasons^11–13^. However, different MWSs lead to different sizes, locations and numbers of the detected clusters in the same data^8,14–16^. Therefore, selecting an appropriate MWS for the SSS is important for accurate cluster identification.

The indicators used to select the MWS mainly include the classic performance measures such as sensitivity, specificity, positive predictive value (PPV), Youden’s index (YDI), misclassification and the recently developed maximum clustering set-proportion (MCS-P) statistic^17^. However, due to the lack of historical prior or true cluster information in practice, these classic performance measures usually cannot be calculated. The MCS-P depends only on the applied spatial data and provides a solution in practice without using any true cluster information or historical prior data. Although for datasets with homogeneous clusters, i.e., clusters having similar relative risks (RR), selecting the MWS with the MCS-P shows good performance compared to that obtained by using the default MWS values, the MCS-P does not perform well for datasets that include significantly heterogeneous clusters^17^. Furthermore, many researchers also use the Gini coefficient to select the maximum reported spatial window size (MRWS) to avoid reporting an overly large cluster and increase the accuracy of the detected clusters^18,19^. The MRWS is the maximum reported population in clusters detected based on a pre-selected fixed MWS larger than the MRWS. Although the Gini coefficient is not used to select the MWS, it can achieve results similar to those obtained by selecting the MWS and it therefore has become a commonly used indicator due to its implementation in the SaTScan software. However, in our exploratory studies, we found that the Gini coefficient may be unstable, is still likely to detect clusters that are much larger than the real clusters, and will even obtain the same results as the 50% MWS, especially for datasets with clusters that are not far from each other, as will be described in detail in the methodology section. Therefore, considering the general existence of complex spatial distribution patterns in reality, especially in health-related datasets, a more efficient indicator is necessary for selecting the MWS.

This study proposed a new indicator based on MCS-P, called the maximum clustering heterogeneous set-proportion (MCHS-P), for selecting an appropriate MWS for the SSS. The MCHS-P can identify potential heterogeneous clusters accurately and depends only on the detected clusters and the applied dataset without any historical prior or true cluster information. Section 2 gives the definition of the MCHS-P and a brief discussion about the limitations and uncertainty for the use of the Gini coefficient that is an alternative indicator for use in practical datasets. Section 3 presents a simulation study and a practical study for the comparison of the MCHS-P with the MCS-P in the selection of MWS for the SSS in different datasets with either homogeneous or heterogeneous clusters. The results obtained using the Gini coefficient and the default 50% MWS are also provided as reference data. Sections 4 and 5 provide a discussion of these results and the conclusion of the paper, respectively.

## Methods

### The SSS and the MCS-P

The SSS is used to identify the likely clusters in spatial datasets. First, it searches for a set of windows under a specified scan parameter. Then, for each window (z), under the alternative hypothesis (z is a cluster) and the null hypothesis (no cluster exists), the logarithm of the likelihood ratio *LLR*(z) is constructed under the Poisson distribution as follows:

$$LLR({\rm{z}})=ln\left\{{\left(\frac{{c}_{z}}{{n}_{z}}\right)}^{{c}_{z}}{\left(\frac{C-{c}_{z}}{C-{n}_{z}}\right)}^{C-{c}_{z}}\right\},$$

where *C* is the total number of the observed cases in the entire study area. ${n}_{z}$ and ${c}_{z}$ are the expected cases under the null hypothesis and the observed cases in z, respectively. Finally, the windows with an *LLR*(z) greater than the critical value obtained from Monte Carlo simulations are selected as the detected significant clusters. Usually, clusters with RR either larger or smaller than 1 are considered for different purposes.

Different MWSs lead to different sets of windows (z) and then different detected clusters. The MCS-P is designed to select the optimal result from the results obtained with different MWSs. First, the MCS-P merges all the detected clusters under a certain single parameter into a union cluster to construct the union *LLR*(z) that reflects the capability of the dataset to support the current result. To get a more stable average value in simulation datasets, the approximately maximal *LLR* based on the most clustering set (MCS) is used to adjust the union *LLR* to the MCS-P. The MCS is composed of all the units with RR either larger or smaller than 1 for the corresponding purpose. Thus, its corresponding *LLR* will be constant in a specified dataset and not necessary in practical application. Such an *LLR* represents the approximately maximal value that the *LLR* can reach when the geographic positions of all the spatial units in the study region are ignored.

1 $$\begin{array}{c}{z}_{i0}={\cup }_{j}{z}_{ij},\\ LLR({z}_{i0})=\,\mathrm{ln}\left\{{\left(\frac{{c}_{{z}_{i0}}}{{n}_{{z}_{i0}}}\right)}^{{c}_{{z}_{i0}}}{\left(\frac{C-{C}_{{z}_{i0}}}{C-{n}_{{z}_{i0}}}\right)}^{C-{c}_{{z}_{i0}}}\right\},\end{array}$$

$${z}_{MCS}=\cup \{x:x\in G,{p}_{x} > {q}_{x}\}$$ or $${z}_{MCS}=\cup \{x:x\in G,{p}_{x} < {q}_{x}\}$$,

$${\rm{MCS}} \mbox{-} {\rm{P}}=\frac{LLR({z}_{i0})}{LLR({z}_{MCS})},$$

where ${z}_{ij}$ is the $j\,$th detected cluster under the *i*th MWS. z_i0_ is the union cluster, *x* is a spatial unit in the entire study area $\,G$, and ${p}_{x}$ and ${q}_{x}$ are the probabilities of an event occurrence in $x$ and out of $x$, respectively. ${z}_{MCS}$ is a window composed of all the units in the MCS. A larger MCS-P means that the corresponding detected clustering set is more supported by the applied dataset.

As observed from Eq. (1), the MCS-P assumes that all the detected clusters under a selected MWS have the same RR. However, in practical datasets such as chronic disease datasets, different clusters may have different RR due to the different environmental and socioeconomic factors. Therefore, the MCS-P may lead to unsatisfactory performance in such datasets, as has been reported in previous studies^17^.

### An improved statistic based on the MCS-P

To improve the performance of the MCS-P in spatial datasets with heterogeneous clusters, the union of detected clusters is reconstructed to fit the potential heterogeneity between the clusters. A very easy approach is to assume that each cluster can have a different RR. However, with a small maximal window size, a single cluster may be detected as a set of small, spatially continuous clusters. Assuming such homogeneous clusters to have different RR may lead to overfitting. Therefore, a constraint is introduced to limit such overfitting. Based on the common knowledge that nonadjacent clusters are usually independent for spatial isolation^20^, the detected clusters under a single certain selected parameter are reconstructed into a set of regions according to their spatial contiguity. Given that the number of detected clusters under the *i*th selected parameter is $y$; then, with only contiguous clusters merged into one clustering region, $y$ detected clusters are merged into *k* $(k\le y)$ merged clusters. The $k$ merged clusters comprise the detected potential heterogeneous clustering set ${S}_{i}(k)$ under the $i\,$th selected parameter. The corresponding union *LLR* $LLR({S}_{i}(k))$ is calculated as:

$$\begin{array}{c}{S}_{i}(k)=\{{z}_{im1},{z}_{im2},\ldots ,{z}_{imk}\},\\ LLR({S}_{i}(k))=\,\mathrm{ln}\left\{{\left(\frac{{c}_{{z}_{im1}}}{{n}_{{z}_{im1}}}\right)}^{{c}_{{z}_{im1}}}{\left(\frac{{c}_{{z}_{im2}}}{{n}_{{z}_{im2}}}\right)}^{{c}_{{z}_{im2}}}\ldots {\left(\frac{{c}_{{z}_{imk}}}{{n}_{{z}_{imk}}}\right)}^{{c}_{{z}_{imk}}}{\left(\frac{C-{\sum }_{j=1}^{k}{c}_{{Z}_{imj}}}{C-{\sum }_{j=1}^{k}{n}_{{z}_{imj}}}\right)}^{C-\mathop{\sum }\limits_{j=1}^{k}{c}_{{z}_{imj}}}\right\}.\end{array}$$

where ${z}_{imj}$ represents the *j*th merged cluster under the *i*th parameter. Such an $LLR({S}_{i}(k))$ takes into consideration both the potential heterogeneity among the detected nonadjacent clusters and the similarity among the spatial contiguous units, and it also limits the overfitting.

To get a more stable average value in simulation datasets, an approximately maximal *LLR* based on the most clustering heterogeneous set (MCHS) is adopted. Unlike for the MCS, the spatial units in the MCHS are permitted to have different probabilities of event occurrence if they are not adjacent. Specifically, the MCS is separated into several subsets based on whether the spatial units are contiguous. The units in the same subset share the same estimated probability of event occurrence. Thus, the units in different subsets may have different probabilities. These heterogeneous subsets compose the MCHS. The corresponding union *LLR* of the MCHS is $LLR({\rm{MCHS}})$.

$$\begin{array}{c}{\rm{MCHS}}=\{{z}_{subset1},{z}_{subset2},\ldots ,{z}_{subsetw}\},\\ LLR({\rm{MCHS}})=\,\mathrm{ln}\left\{\left(\mathop{\prod }\limits_{j=1}^{w}{\left(\frac{{c}_{{z}_{subsetj}}}{{n}_{{z}_{subsetj}}}\right)}^{{c}_{{z}_{subsetj}}}\right){\left(\frac{C-{\sum }_{j=1}^{w}{c}_{{Z}_{subsetj}}}{C-{\sum }_{j=1}^{w}{n}_{{z}_{subsetj}}}\right)}^{C-\mathop{\sum }\limits_{j=1}^{w}{c}_{{Z}_{subsetj}}}\right\},\end{array}$$

where $w$ is the number of subsets in the MCHS and ${z}_{subsetj}$ represents the window composed of the spatial units in the jth subset. The denominator $LLR({\rm{MCHS}})$ represents the approximately maximal union *LLR* in the specified *G* with potential heterogeneous nonadjacent clusters, which is a constant in a specified dataset and not necessary in practical application. Then, the improved indicator MCHS-P is given as:

$${\rm{MCHS}} \mbox{-} {\rm{P}}=\frac{LLR({S}_{i}(k))}{LLR({\rm{MCHS}})}.$$

The MCHS-P reflects the ratio of the union *LLR* between the detected potential heterogeneous clustering set and the most clustering heterogeneous set in *G*. With the detected clusters reconstructed into a potential heterogeneous clustering set, the MCHS-P adapts to spatial datasets with homogeneous clusters and heterogeneous clusters and is still an approximate relative indicator that depends only on the detected clusters and the applied dataset rather than on any historical prior or true cluster information.

### The Gini coefficient for MRWS selection

**Figure 1:**
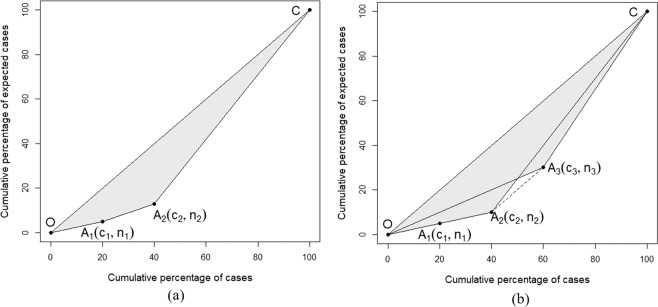
Illustration of the Gini coefficients. Subfigure (**a**) is for a cluster model with two clusters (A1 and A2). Subfigure (**b**) is a comparison between the two Gini coefficients of the two detected results. The first coefficient is for two homogeneous small clusters (A1 and A2), and the second is for a large cluster (A3).

To select the MRWS, the Gini coefficient measures the difference of the case distribution in the clusters and non-clusters based on the cumulative cases and expected cases in the clusters. First, the clusters detected under a certain MRWS are listed in descending order by RR. Second, the cumulative percentage of cases and the cumulative percentage of expected cases for each cluster point are computed. For example, as shown in Fig. 1(a), points O and C are (0, 0) and (100, 100), respectively, so that the reciprocal of the slope of OC measures the expected RR, i.e., $\text{RR}\,=\,1$. Two clusters (A_1_ and A_2_) are detected and the reciprocal of the slope of OA_1_ is larger than A_1_A_2_, meaning that the RR of A_1_ is larger than A_2_. c_1_ and n_1_ are the observed and expected cases in cluster A_1_, respectively; c_2_ is the sum of the observed cases in A_1_ and A_2_ and n_2_ is the sum of the expected cases in A_1_ and A_2_. The Gini coefficient is equal to two times the area of OA_1_A_2_C, namely, $Gini({{\rm{A}}}_{1}{{\rm{A}}}_{2})=2\times {S}_{{{\rm{OA}}}_{1}{{\rm{A}}}_{2}{\rm{C}}}$. Finally, the MRWS with the largest Gini coefficient is selected as the optimal MRWS, as shown in detail by Han *et al*.^18^.

Although the Gini coefficient provides a better detected result for the SSS than the default MWS in many situations, in our exploratory studies, we found that the improvement may be unstable and insignificant, especially for datasets with homogeneous clusters that are not far from each other. A typical case is shown in Fig. 1(b), where the Gini coefficients for the two detected results are presented. The first detected result includes two real homogeneous small clusters (A_1_ and A_2_) and the other includes a large detected cluster A_3_ that is a superset of A_1_ and A_2_. When the incidence of these spatial units in A_3_ but out of A_1_ and A_2_ is equal to the expected RR, line A_2_ A_3_ is parallel to OC and the area of OA_2_A_3_ is equal to that of CA_2_A_3_, namely, $Gini({{\rm{A}}}_{1}{{\rm{A}}}_{2})=Gini({{\rm{A}}}_{3})$. Thus, a small random fluctuation of RR in the spatial units in A_3_ but out of A_1_ and A_2_ will lead to a smaller slope of A_2_A_3_ than that of OC. As a result, $Gini({{\rm{A}}}_{1}{{\rm{A}}}_{2})$ will be smaller than $Gini({{\rm{A}}}_{3})$ and A_3_ will be falsely detected as clusters.

Therefore, selection of the MRWS using the Gini coefficient may not obtain good results in the complex practical datasets. Nevertheless, this approach is still commonly used owing to its convenient implementation in the SaTScan software.

## Result

### Simulation study

To validate the capability of the MCHS-P to select an appropriate MWS for the SSS in datasets with heterogeneous clusters, we employed a series of simulation datasets with different heterogeneities among the clusters to compare the performance of the MCHS-P with that of the MCS-P. Additionally, to evaluate the risk of the MCHS-P selecting worse parameters than the MCS-P because of overfitting in datasets with homogeneous clusters, we also used a series of simulation scenarios with different random fluctuations out of the homogeneous cluster, in which a higher random fluctuation is more likely to lead to a false detected cluster. The results with the default 50% MWS and Gini coefficient were also provided as reference data.

#### Simulation data

Simulation datasets are taken from Kulldorff’s benchmark data^21^ that have been commonly used for the evaluation of SSS with different scan parameters^22–24^ and the validation of the MCS-P in selecting MWS^17^. These simulation datasets are based on a real dataset for breast cancer mortality during 1988–1992 that consists of the data for 29,535,210 women in 245 counties in the northeastern USA.

Two total case numbers, 600 and 6000, were considered. For each number, three different sets of concentric circle clusters were constructed that included women located in three different areas, such as rural, mixed, and urban areas. Each of these three sets contains clusters with different sizes, for example, 1, 2, 4, 8, or 16 counties. Based on these clusters, a total of 50 simulation scenarios were built. The details are shown in Table 1. For each scenario, 10,000 datasets were generated. All the datasets are available at the SaTScan website^25^.

**Table 1:** Simulation scenarios with homogeneous/heterogeneous clusters.

Location		Homogeneous clusters									Heterogeneous clusters			
		Rural			Mixed			Urban			Two clusters		Three clusters	
Cases	Size	POP	RR	P	POP	RR	P	POP	RR	P	POP	H	POP	H
600	1	2675	192.89	1.998	710196	2.85	1.946	786178	2.73	1.941	788853	190.16	1499049	190.16
	2	22911	27.03	1.992	817050	2.70	1.943	1072181	2.43	1.932	1095092	24.6	1912142	24.6
	4	132343	7.05	1.979	1108440	2.40	1.931	2953077	1.81	1.881	3085420	5.24	4193860	5.24
	8	204829	5.35	1.971	1352284	2.24	1.923	5018909	1.63	1.836	5223738	3.72	6576022	3.72
	16	360275	3.9	1.961	1684327	2.1	1.914	7627173	1.53	1.785	7987448	2.37	9671775	2.37
6000	1	2675	23.73	20.27	710196	1.45	20.09	786178	1.43	20.08	788853	22.3	1499049	22.3
	2	22911	4.96	20.25	817050	1.42	20.09	1072181	1.36	20.05	1095092	3.6	1912142	3.6
	4	132343	2.21	20.21	1108440	1.36	20.04	2953077	1.22	19.88	3085420	0.99	4193860	0.99
	8	204829	1.92	20.18	1352284	1.32	20.02	5018909	1.17	19.73	5223738	0.75	6576022	0.75
	16	360275	1.66	20.15	1684327	1.29	19.99	7627173	1.15	19.57	7987448	0.51	9671775	0.51

Note: POP is the total population in the clusters. RR is the relative risk of the clusters. P is the incidence (×10^−5^) out of the clusters. Because the case numbers follow a Poisson distribution under the null hypothesis, the incidence rate also reflects random fluctuation out of the clusters. H is the difference between the maximal RR and minimal RR that reflects the strength of the heterogeneity among the clusters. Heterogeneity among the clusters becomes lower as the cluster number, total case number and cluster size grow.

These 50 scenarios include 10 simulation scenarios with two heterogeneous equal-sized clusters located in the rural and urban areas, respectively; 10 scenarios with three heterogeneous equal-sized clusters located in the rural, mixed and urban areas, respectively; and 30 scenarios with a single cluster that can be deemed as scenarios with homogeneous clusters, since there is no heterogeneity for a single cluster. For the heterogeneous cluster models, the scenarios with a smaller cluster size, two clusters and 600 cases are more heterogeneous than those with a larger cluster size, three clusters and 6,000 cases, respectively.

These scenarios are referred to as “total case numbers-cluster location-cluster size” for short. For example, 6000-rural-1 refers to the scenario with a total case number of 6,000, one cluster located in the rural area, and one county for the cluster; 6000-two-4 refers to the scenario with a total case number of 6,000, two clusters located in the rural and urban areas, and four counties for each cluster.

**Figure 2:**
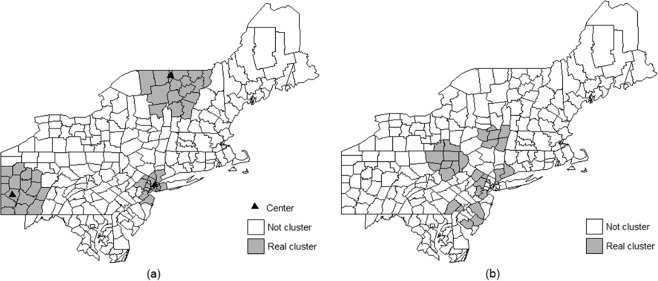
Positions of real clusters. Subfigure (**a**) is the position of real clusters with 16 counties for each in Kulldorff’s benchmark datasets and the solid triangles are the centres for the three types of clusters such as those located in rural, mixed and urban areas. Subfigure (**b**) shows the positions of the real clusters in the additional scenario in which the clusters are not far away from each other.

In addition, as shown in Fig. 2(a), the three clusters with different centres in the above-described scenarios are so far away from each other, for example with the away region containing more than 50% of the total population at risk, that even the 50% MWS could not cover any two clusters and therefore, the false positive rate will be underestimated in such datasets. Considering that the clusters that are not located so far apart are common in practical datasets, we built an additional scenario that is similar to the complex real datasets, called the complex scenario available from Supplementary File 1, in which the artificial clusters are not located far apart, as shown in Fig. 2(b). In the complex scenario, a total of 6,000 cases were included and the RR in these clusters were set to 1.55.

#### Performance comparison

From 50 different MWSs in the range of 1–50% with an interval of 1% population at risk, the MCHS-P and the MCS-P were used to select their own optimal MWS under the most commonly used circle window. Only significant clusters with a P-value less than 0.05 and no geographic overlap with more likely clusters were considered; these parameters were also set for the detected result obtained from the Gini coefficient and the 50% MWS. The SaTScan9.4 free software was selected as the clustering detection tool.

Subsequently, MWSs with the largest the MCHS-P and the MCS-P were selected as their own optimal parameters, respectively. The SSS’s performance characteristics obtained using the MCHS-P, MCS-P, 50% MWS, and Gini coefficient were compared using the average values of 5 commonly used classic performance measures over replicas: sensitivity, specificity, positive predictive value (PPV), Youden’s index (YDI) and misclassification^22,26–29^. Additionally, we calculated the average values of the MCHS-P, the MCS-P and each classic performance measure for each MWS over replicas to evaluate the relationships of the MCHS-P and the MCS-P with the classic performance measures.

Of these five classic performance measures, sensitivity reflects the capacity to correctly identify true clusters. Specificity and PPV reflect the capability to correctly identify the units out of clusters. YDI and misclassification reflect both aspects. Because the measures based on the population may be more robust^22^, the five classic performance measures were calculated as follows:

$$\begin{array}{c}{\rm{sensitivity}}=\frac{a}{a+b},\\ {\rm{specificity}}=\frac{d}{d+c},\\ {\rm{PPV}}=\frac{a}{a+c},\\ {\rm{YDI}}={\rm{sensitivity}}+{\rm{specificity}}-1,\\ {\rm{misclassification}}=\frac{b+c}{a+b+c+d},\end{array}$$

where a, b, c and d represent the total population in the four types of spatial units (units in true clusters and detected clusters, units in true clusters and not in detected clusters, units in detected clusters and not in true clusters and units in neither detected clusters nor true clusters, respectively).

**Figure 3:**
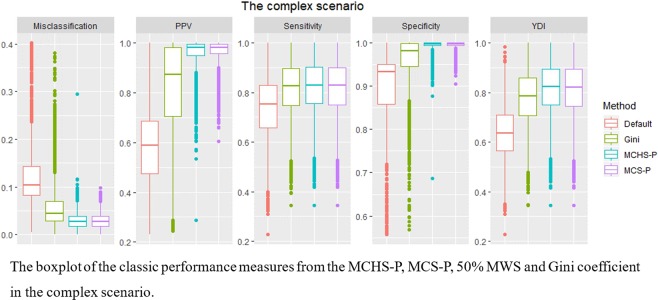
Comparison of the results between the MCHS-P, MCS-P, 50% MWS and Gini coefficient in the complex scenario. It is observed that the MCS-P and the MCS-P have more stable and better performance than the 50% MWS and the Gini coefficient.

**Figure 4:**
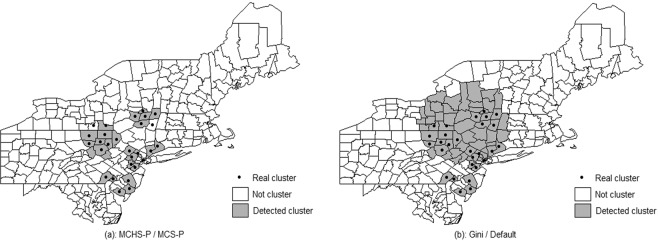
Comparison between the MCHS-P, the MCS-P, the default MWS and the Gini coefficient in one of simulated datasets in the additional scenario with a complex cluster model. Subfigure (**a**) is the detected result under the 2% MWS selected by both the MCHS-P and the MCS-P. Subfigure (**b**) is the detected result under the 50% MRWS selected by the Gini coefficient and the default 50% MWS.

Comparison between the four methods. The average sensitivity, specificity, PPV, YDI and misclassification over replicas from the MCHS-P, MCS-P, 50% MWS, and Gini coefficient were compared, and the detail is seen in Supplementary File 2. Generally, in 50 types of benchmark datasets, the 50% MWS and the Gini coefficient performs well but slightly poorer than the MCHS-P, especially in the scenarios with multiple clusters or clusters covering large population. However, in the complex scenario, as shown in Figs. 3 and 4, the default 50% MWS performs poorly due to its high false positive rate. The Gini coefficient makes a mild and unstable improvement, while the MCHS-P still has a consistently good performance. The MCS-P exhibits the same performance as found in the previous study, that is to say its performance is good in scenarios with homogeneous clusters and is poor in scenarios with heterogeneous clusters, especially for the highly heterogeneous clusters such as 600-two-1, 600-two-2, 600-three-1, 6000-two-1, and 6000-three-1. As expected, the MCHS-P has much improvements in the heterogeneous scenarios and maintains similar performance with the MCS-P in the homogeneous scenarios.

Specifically, in the 20 scenarios with heterogeneous clusters (multiple clusters), as the heterogeneity increases, corresponding to the decrease in the cluster size, the total case number and cluster number, the MCHS-P become more advantageous compared to the MCS-P, and the largest improvements in sensitivity, YDI and misclassification reach 540%, 538% and 69.5% (0.9047 vs 0.1413, 0.9000 vs 0.1410 and 0.0071 vs 0.0232), respectively, in 600-two-1, whereas the specificity and PPV values decline slightly by 0.44% and 4.58% (0.9953 vs 0.9997 and 0.9442 vs 0.9895), respectively. Although the 50% MWS and the Gini coefficient also show a good performance for the large separation between clusters, they are still outperformed by the MCHS-P. An examination of the data presented in Table 2 shows that the MCHS-P achieves optimal aggregative indicators such as YDI and misclassification in all 20 scenarios. In the 30 scenarios with homogeneous clusters (a single cluster), the overall average values of the sensitivity, specificity, PPV, YDI and misclassification under the parameter selected using the MCHS-P show only a very slightly inferior values compared to the MCS-P (0.0002, 0.0005, 0.0068, and 0.0007 smaller and 0.0005 larger, respectively). Although the disadvantage of the MCHS-P relative to the MCS-P increases as the total case number and cluster size decrease, the largest disadvantages in the sensitivity, specificity, PPV, YDI and misclassification found in 600-rural-1 are still very small (0.0000, −0.0024, −0.0524, −0.0024, and 0.0024, respectively). Compared with the 50% MWS and the Gini coefficient, although the performance of the MCHS-P is poorer in several scenarios with a single high-RR and low-population cluster, the maximal difference is still very small, for example, 0.0007, for both YDI and misclassification found in 600-rural-1. In addition, as shown in Table 2, both the MCHS-P and the MCS-P obtain the optimal result for both YDI and misclassification in more than 20 out of 30 scenarios, while the Gini coefficient obtains the optimal value for YDI in only 2 scenarios.

**Table 2:** Frequency of the MCHS-P, MCS-P, 50% MWS, and Gini coefficient obtaining the optimal result* in 20 multi-clusters scenarios and in 30 single-cluster scenarios for the five classic performance measures.

Scenario type	In 20 multi-cluster scenarios				In 30 single-cluster scenarios			
Measures	MCHS-P	MCS-P	Default	Gini	MCHS-P	MCS-P	Default	Gini
Sensitivity	19	0	1	0	26	28	4	4
Specificity	0	20	0	0	26	30	26	26
PPV	0	20	0	0	2	5	25	26
YDI	20	0	1	1	22	28	2	2
Misclassification	20	0	4	6	26	30	22	22

^*^The optimal result is defined as the range that varies within 0.001 around the real optimal value of classic performance measure, such as the maximal sensitivity, specificity, PPV, YDI and the minimal misclassification. Therefore, in a given scenario, it is possible for more than one method to obtain the optimal result.

In the complex scenario, the 50% MWS performs poorly for detecting an overly large cluster including several small real clusters, in agreement with the previous study^6,17,30^. As shown in Table 3, the Gini coefficient improves the performance, reaching the values of 0.0780, 0.0647, 0.2222, 0.1428, and 0.0665 for sensitivity, specificity, PPV, YDI, and misclassification, respectively. Compared with the MCS-P and the MCHS-P, which have similar performance for the homogeneity between the clusters, the improvement due to the use of the Gini coefficient is still insufficient, e.g., on average, the MCHS-P and the MCS-P reduce 76.7% misclassification relative to the default MWS, while the reduction due to the Gini coefficient is 54.2%; additionally, the performance of the Gini coefficient method is much more unstable than those of the MCHS-P and the MCS-P, especially for misclassification, PPV and specificity, as shown in Fig. 3. A further examination of the simulated datasets shows that the Gini coefficient selected 50% MRWS as optimal in some cases and leads to the same detected results as the default MWS, giving high false positive rates. Figure 4 shows one such dataset out of the 10,000 simulated datasets in which the detected large cluster does indeed include the non-cluster region between the several small real clusters, further supporting the discussion of the Gini coefficient in the methodology section above.

**Table 3:** Average classic performance measures over replicas from the MCHS-P, MCS-P, 50% MWS and Gini coefficient in the complex scenario.

Measures	MCHS-P	MCS-P	Default	Gini
Sensitivity	0.8207	0.8173	0.7365	0.8145
Specificity	0.9944	0.9955	0.8989	0.9637
PPV	0.9611	0.9672	0.6059	0.8281
YDI	0.8151	0.8128	0.6354	0.7782
Misclassification	0.0287	0.0282	0.1226	0.0561

#### The relationship of the MCHS-P and the MCS-P with classic performance measures

In addition to the comparison of optimal MWS selected using the MCHS-P, MCS-P, Gini coefficient and default MWS, the relationships between the MCHS-P, MCS-P and classic performance measures such as sensitivity, specificity, PPV, YDI and misclassification, were evaluated to validate whether the selection of MWS using the MCHS-P can generally lead to detected results with better spatial accuracy, seen in Supplementary File 3.

Generally, as found previously, a larger MCS-P is related to better classic performance measures in scenarios with homogeneous and weakly heterogeneous clusters but is related to poorer performance in scenarios with heterogeneous clusters. By contrast, the MCHS-P is consistently positively related to the performance measures in all scenarios. Consistently high specificity and PPV were found in all scenarios similar to previous work^17^.

**Figure 5:**
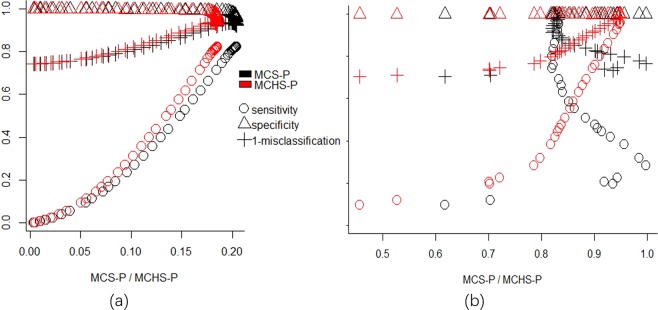
Relationship of the MCHS-P and the MCS-P with classical performance measures. Subfigure (**a**) is for a homogeneous scenario such as 6000-urban-16, and shows that both the MCHS-P and the MCS-P have a positive relationship with the classic performance measure. Subfigure (**b**) is for a heterogeneous scenario such as 6000-three-16RR3.0-2.0-1.2, and it shows that the MCHS-P has a positive relationship with the classic performance measures, whereas the MCS-P has a nearly negative relationship with these performance measures.

Several typical scenarios were selected for a detailed demonstration of the relationship of the MCHS-P and the MCS-P with classic performance measures and the difference between the MCHS-P and the MCS-P. In scenarios with homogeneous clusters, 6000-urban-16 was selected for a clear demonstration because its large population in clusters make the detected result sensitive to the MWS. Because PPV and YDI are almost equal to specificity and sensitivity in this scenario, respectively, only specificity, sensitivity and accuracy (1-misclassification) were presented. As shown in Fig. 5(a), both the MCHS-P and the MCS-P show positive relationships with sensitivity and accuracy, meaning that both the MCHS-P and the MCS-P will select the MWS with the optimal result when the clusters are homogeneous.

In scenarios with heterogeneous clusters, 600-two-1 was selected for its high heterogeneity between the clusters. Because the small population in the clusters makes it difficult to clearly present the relationship using a graphic plot, detailed values of the MCS-P, MCHS-P and performance measures were provided, shown in Supplementary File 2. the MWS with the largest the MCS-P has the lowest sensitivity, YDI and highest misclassification, whereas the MWS with the largest MCHS-P has the highest sensitivity, YDI and lowest misclassification. The specificity and PPV always stay at a rather high level and show little variation. On the other hand, because the benchmark datasets with large population in clusters have weak heterogeneity, for example 6000-three-3 and 600-three-3 shown in Table 1, we built an additional simulation scenario, available from Supplementary File 4, including clusters with relatively high heterogeneity and larger population to present the detailed relationship more clearly using a graphic plot. In this scenario, the cluster model has the same artificial clustering regions, the population at risk and total case number as those of 6000-three-16. Differently, the heterogeneity among the clusters was increased by using RRs of 3.0, 2.0 and 1.2 for the clusters located in rural, mixed and urban areas. The differences in RR between the clusters are much smaller than those in scenarios with simulated heterogeneous clusters, for example 190.16 in 600-two-1 and 2.37 in 600-two-16. This new scenario is abbreviated as 6000-three-16RR3.0-2.0-1.2. Similar to 6000-urban-16, PPV and YDI are also almost equal to the specificity and sensitivity, respectively. As shown in Fig. 5(b), the relationship of the MCS-P and the MCHS-P with classical performance measures shows that in scenarios with high heterogeneity, the MCHS-P showed a positive association with sensitivity and accuracy, whereas the MCS-P shows a negative association with these measures. This means that in the datasets with highly heterogeneous clusters, the MCHS-P still can select the optimal MWS while the MCS-P fails. Therefore, given the stable positive association to classic performance measures in both scenarios with homogeneous and heterogeneous clusters, a detected result with higher MCHS-P will also have better classic performance measures suggesting better spatial accuracy.

### Practical study: comparison between the four methods

To provide examples of application of the MCHS-P, we employed two real datasets to compare the difference between the detected clusters obtained using the default parameters, selected by the MCHS-P, MCS-P, and Gini coefficient. The first data set is for female breast cancer which is a chronic disease and the second dataset is for measles which is an infectious disease that was used in a previous study^17^.

#### Female breast cancer mortality in the northeastern USA

The recent breast cancer dataset that is the same as those in the simulation study was obtained from the official website of the National Centre for Health Statistics and includes a total of 44,182 female deaths from breast cancer in 245 counties in the northeastern USA in 2011–2015^31^. The annual average total female population is 32,587,167. Unlike the artificial clusters in simulated datasets, the real dataset shows more complex spatial pattern that reflects the characteristics of the breast cancer which is a chronic disease.

For the more complex characteristics in this practical dataset, 500 MWSs in the 0.1–50% range with an interval of 0.1% population at risk were set as the candidate parameters in order to obtain a more accurate result. Other settings are the same as those in the simulation study.

**Figure 6:**
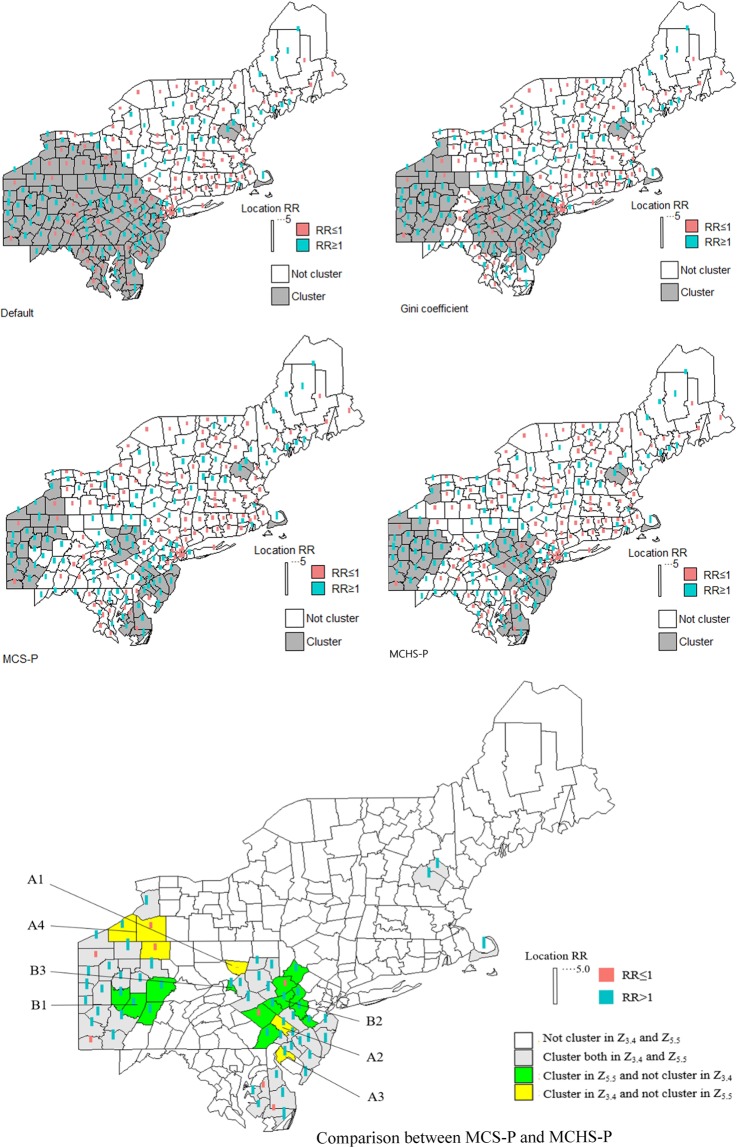
Detected clusters obtained using different parameters. The top four subfigures are for the default MWS (50%) and 30% MRWS selected by the Gini coefficient, the 3.4% MWS selected by the MCS-P and the 5.5% MWS selected by the MCHS-P. The bottom subfigure shows the comparison between the results from the MCHS-P and the MCS-P.

The 3.4% and 5.5% MWSs were selected using the MCS-P and the MCHS-P, respectively and the 30% MRWS was selected using the Gini coefficient. The detected results obtained using the MCHS-P, MCS-P, default MWS (50%) and Gini coefficient are shown in Fig. 6. The four detected results mainly differ in the southwestern region. With the default 50% MWS, the SSS detected a large cluster covering over 45% of the total female population. Large number of low-mortality counties were included and led to an average RR of 1.1. The Gini coefficient shows better performance but still detected large regions as clusters in which the proportion of the female population reach 38% and the average RR is 1.12. With limited public health resources, such a large detected cluster with flat RR may not provide accurate information for further interventions. The MCHS-P and the MCS-P tend to detect the clusters with smaller female populations, such as 22.6% and 20.0%, and higher RR, such as 1.20 and 1.22, respectively.

Comparing the results of the MCS-P and the MCHS-P, a total of 50 and 58 counties were detected as clusters using the MWSs of 3.4% (Z_3.4_) and 5.5% (Z_5.5_), respectively. Of these counties, 44 are identical. Table 4 shows that six counties were detected using Z_3.4_ but were not detected using Z_5.5_, and three of these had RR values smaller than 1. Fourteen counties were detected using Z_5.5_ but were not detected using Z_3.4_, and only two of them had RR values smaller than 1. Figure 6 shows that the heterogeneity among the detected clusters is not high and the different counties detected as clusters using Z_3.4_ and Z_5.5_ are distributed around the identical counties. The eastern four counties labelled as B1 with relatively high mortality were not detected as clusters by Z_3.5_, even though all of their eastern neighbouring counties were detected as clusters, suggesting that possible clusters were omitted using Z_3.5_. By contrast, they were detected by Z_5.5_. The counties labelled B2 and B3 display a similar performance to that of B1. The regions labelled A1 and A4 with low mortality were detected as clusters by Z_3.4_, suggesting incorrectly reported low-mortality regions. By contrast, they were excluded by Z_5.5_. Although A2 and A3 with a relatively high mortality were omitted by Z_5.5_, A2 and A3 include only two counties, and the heterogeneity among the clusters is low.

**Table 4:** Different counties detected using 3.4% and 5.5% MWSs selected using the MCS-P and the MCHS-P.

Found only in the MCS-P/MCHS-P	Region	Code	Pop	RR
MCS-P	A1	42113	3038	**0.01**
	A2	42091	41794	1.116985
	A3	34033	33325	1.306212
	A4	36009	39828	**0.888781**
		36013	66959	1.10174
		42083	20874	**0.953987**
MCHS-P	B1	42005	34275	1.248418
		42021	70657	1.284748
		42033	38982	1.21123
		42063	43982	1.09015
	B2	34019	63792	1.005898
		34021	189129	1.002259
		34041	55027	1.139572
		42011	210473	**0.988145**
		42029	258927	1.074539
		42077	183185	1.144406
		42089	84994	**0.910956**
		42095	152641	1.063357
		42103	28268	1.148192
	B3	42093	9582	1.30857

Note: RR values less than 1 are highlighted in bold.

#### Measles incidence in henan province, china

**Figure 7:**
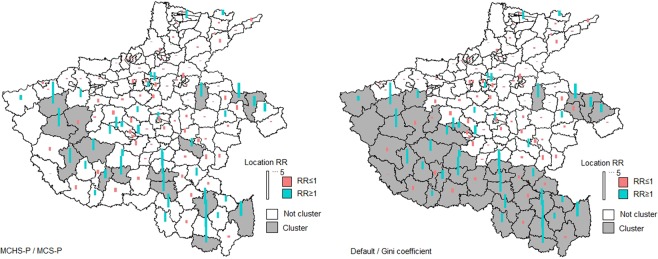
Detected clusters obtained using different parameters. The left subfigure is for the results obtained using 1.6% MWS selected by the MCHS-P and the MCS-P, and the right subfigure is for the results obtained using the default MWS (50%) and the MRWS (20–50%) selected by the Gini coefficient.

The measles incidence data in Henan Province, China in May 2009 that was used to validate the MCS-P in selecting the MWS^17^ was obtained from the disease reporting system of China CDC. A total of 1,371 measles cases were reported in a population of 91,669,661. The four methods, i.e., the MCHS-P, MCS-P, default MWS and Gini coefficient, had the same parameter set as that in the female breast cancer mortality dataset discussed above. As shown in Fig. 7, the default MWS (50%) and the Gini coefficient obtained the same result and both detected a large region as clusters in which the proportion of the population to the total population reaches 34.7% but a low RR such as 1.96. the MCHS-P and the MCS-P selected the same MWS (1.6%) due to the weak heterogeneity between the clusters. They detected several small clusters with a 12.0% proportion of population and a high RR of 3.26, obviously decreasing the false positive rate by excluding the counties with lower RR.

## Discussion

Both the practical study and simulation study suggested that the detected result of the SSS is highly sensitive to the MWS. The commonly used default 50% MWS reports relatively accurate clusters in dataset with clusters far away from each other, but reports an overly large cluster in datasets with clusters that are not far away, leading to a high false positive rate so that it can hardly provide accurate results. Although without any prior information, the Gini coefficient can improve the detected result by selecting a MRWS, but it is still likely to report a cluster that is larger than the real cluster. In particular, in datasets with complex cluster distributions, the Gini coefficient even has the same poor result as the default MWS. Although the MCS-P can select an appropriate MWS to avoid reporting such large clusters and improves the accuracy of the results in practical datasets, heterogeneous clusters in the applied spatial datasets may lead to unsatisfactory performance. In such cases, the modified MCHS-P showed better performance than the MCS-P in both simulation and practical datasets with heterogeneous clusters. Meanwhile, it showed no significant difference from the MCS-P by assuming a similar probability of event occurrence in contiguous clusters in order to limit overfitting. As the heterogeneity between clusters increases, the advantage of the MCHS-P over the MCS-P becomes even greater.

Similar to previous studies, in the scenarios with clusters very far away, specificity and PPV stay at high level^6,17,27,32^, so that the sensitivity and overall performance measures such as YDI and misclassification are of more interest in most studies. In all simulation scenarios with heterogeneous clusters, the values of sensitivity, YDI and misclassification under the parameter selected using the MCHS-P were greatly improved compared to those obtained using the MCS-P. Moreover, higher heterogeneity leads to greater improvement of up to 540.27%, 537.92% and 69.40%, for sensitivity, YDI and misclassification, respectively. The correlation of the MCHS-P and the MCS-P with classical performance measures (Fig. 5) shows that in the datasets with little heterogeneity among the clusters, the results detected with a higher MCS-P and MCHS-P have better sensitivity, accuracy and YDI. Additionally, as the heterogeneity increases, the MCHS-P is still positively related to sensitivity, accuracy and YDI, whereas the relationship of the MCS-P with them becomes negative. Therefore, an appropriate parameter can be selected using the MCHS-P in spatial datasets with highly heterogeneous clusters for which the MCS-P will fail. In simulation datasets with homogeneous clusters, the performance of the MCHS-P is disadvantageous in some cases compared to the MCS-P because the MCHS-P may mistakenly identify high-risk regions caused by random fluctuation as a cluster for overfitting. However, the average disadvantage values for sensitivity, specificity, PPV, YDI, and misclassification are only 0.0002, 0.0005, 0.0068, 0.0007 and 0.0005, respectively. Although the disadvantage increases with the decrease in the total case and cluster size because false clusters can be more easily detected in datasets with small cluster sizes and total case number, the largest disadvantage remains very slight (0.0000, −0.0024, −0.0524, −0.0024, and 0.0024, respectively). These results suggest that assuming the similarity of contiguous clusters effectively limited overfitting in the MCHS-P and obtained a similar result with the MCS-P in spatial datasets with fairly homogeneous clusters.

Compared to the commonly selected default 50% MWS and the Gini coefficient, the MCHS-P shows better performance in most scenarios. As the population in clusters and the number of clusters increase, meaning that the random fluctuation decreases, the advantage of the MCHS-P over the Gini coefficient and the default parameter increases, and especially in the additional simulation scenario with the complex spatial pattern, the average advantage values over the default MWS were 11.4%, 10.6%, 58.6%, 28.3%, 76.7% for sensitivity, specificity, PPV, YDI and misclassification, respectively, with the corresponding values of 0.7%, 3.1%, 16.1%, 4.7%, and 48.9% over the Gini coefficient. In addition, the improvement due to the use of the Gini coefficient was found to be unstable, especially for misclassification, PPV and specificity in complex datasets. Only in the scenarios with high-RR and low-population single cluster does the MCHS-P have a slightly worse performance, for instance with the maximal difference for both misclassification and YDI of only 0.0007 observed in rural-600-1 as presented in Supplementary File 2.

In the case study of female breast cancer mortality data, most counties in the clusters detected using MWSs selected by the MCS-P and the MCHS-P are identical. Although the heterogeneity among the clusters is not high, the SSS’s performance under the MWS selected using the MHCS-P is still better than that of the MCS-P. As the case result shows, the MCHS-P selected a larger MWS than the MCS-P did because the MCHS-P assumes that nonadjacent clusters are independent and each cluster can incorporate more neighbour counties for which the mortalities are closer to this cluster and are far away from other clusters. Therefore, more counties with a relatively high RR that are close to the clusters detected using the MCS-P are detected using the MCHS-P. In the two practical datasets, the Gini coefficient and the default MWS both reported clusters including many low-risk spatial units, which may provide poor information in fields with limited resources such as public health. Although the Gini coefficient led to some improvements in the first practical dataset, it made no improvements in the second practical dataset. Such unstable improvements of the Gini coefficient were also found in the complex scenario.

Besides, as we stated in the method section, since the denominator of MCS-P and MCHS-P is a constant regardless of the selected MWS, it is not necessary to calculate the denominator of MCS-P and MCHS-P when we use them to select the MWS in a practical dataset.

## Conclusion

Most practical datasets may contain multiple clusters that are not so far away from each other, and the SSS with default MWS is much more likely to detect overly large clusters with poor accuracy. In this case, selecting an appropriate MWS is critical for the accurate performance of the SSS. Although the Gini coefficient and the MCS-P can be used to select a more appropriate MRWS or MWS to improve the detected result, the Gini coefficient may be unstable and obtain insignificant improvements. The MCS-P will select an inappropriate MWS in the dataset with highly heterogeneous clusters. By contrast, the improved MCHS-P will have stable and good performance. Additionally, as found for the relationship between the MCHS-P and the classic performance measures, results detected with a higher MCHS-P will show good performance for sensitivity, specificity, PPV, YDI and misclassification regardless of the homogeneity and heterogeneity between the clusters. Therefore, in the case without prior regarding true clusters or with the prior of heterogeneous clusters, the MCHS-P should be recommended for selecting an appropriate MWS for the SSS to obtain better accuracy. In the case with the prior that only homogeneous clusters exist, MCS-P should be employed to achieve best performance which has slight advantages over that using MCHS-P.

## Supplementary information

The detailed simulation results in Kulldorff's benchmark datasets.

Dataset 1.

Dataset 2.

Dataset 3.

## Data Availability

• The simulation datasets used during this study are available on the website of SaTScan for the benchmark datasets: https://www.satscan.org/datasets/nebenchmark/index.html and the Supplementary Files 1 and 4 for the complex scenario and 6000-three-16RR3-2-1.2, respectively. • The breast cancer dataset in the case study is available on the official website of the National Centre for Health Statistics: https://www.cdc.gov/cancer/uscs/dataviz/download_data.htm. And the measles incidence dataset is available from the corresponding author on reasonable request.
