# Supplementary material for: Using the maximum clustering heterogeneous set-proportion to select the maximum window size for the spatial scan statistic
Source: Sci Rep. 2020 Mar 17;10:4900. doi: 10.1038/s41598-020-61829-y (PMC7078301; doi:10.1038/s41598-020-61829-y)
Supplement: Supplementary file 1 — The detailed simulation results in Kulldorff's benchmark datasets. [file 41598_2020_61829_MOESM1_ESM.docx]

**Using the maximum clustering heterogeneous set-proportion to select the maximum window size for the spatial scan statistic**

Wei Wang^1, †^, Tao Zhang^1, †^, Fei Yin^1^, Xiong Xiao^1^, Shiqi Chen^2^, Xingyu Zhang^3^, Xiaosong Li^1^, Yue Ma^1^*

1 West China School of Public Health and West China Fourth hospital, Sichuan University.

2 Women and Children's Health Management Department, Sichuan Provincial Hospital for Women and Children

3 Department of Systems, Populations and Leadership, University of Michigan, School of Nursing

^†^ These authors contributed equally to this work.

* Correspondence to [gordonrozen@qq.com](mailto:gordonrozen@qq.com).

Table 1 Average values of classic performance characteristics over replicas from the MCHS-P, MCS-P, 50% MWS, and Gini coefficient in scenarios with several heterogeneous clusters

| Size | Cases | 600 | | | | | | | | |  |  | 6000 | | | | | | | |
| --- | --- | --- | --- | --- | --- | --- | --- | --- | --- | --- | --- | --- | --- | --- | --- | --- | --- | --- | --- | --- |
|  | Cluster location | Rural and urban | | | |  | Rural, mixed and urban | | | |  | Rural and urban | | | |  | Rural, mixed and urban | | | |
|  | Measures | MCHS-P | MCS-P | Default | Gini |  | MCHS-P | MCS-P | Default | Gini |  | MCHS-P | MCS-P | Default | Gini |  | MCHS-P | MCS-P | Default | Gini |
| 1 | Sensitivity | **0.9047** | **0.1413** | **0.9015** | **0.9013** |  | **0.8684** | **0.4282** | **0.8511** | **0.8505** |  | **0.8631** | **0.1867** | **0.8181** | **0.8169** |  | **0.8345** | **0.5024** | **0.7825** | **0.78** |
|  | Specificity | **0.9953** | **0.9997** | **0.9955** | **0.9955** |  | **0.9934** | **0.9981** | **0.9935** | **0.9936** |  | **0.993** | **0.9992** | **0.9934** | **0.9935** |  | **0.9919** | **0.9971** | **0.992** | **0.9922** |
|  | PPV | **0.9442** | **0.9895** | **0.9493** | **0.9493** |  | **0.9094** | **0.9704** | **0.913** | **0.9134** |  | **0.9245** | **0.9824** | **0.9356** | **0.9368** |  | **0.8945** | **0.9567** | **0.8987** | **0.9003** |
|  | YDI | **0.9001** | **0.1411** | **0.897** | **0.8969** |  | **0.8617** | **0.4263** | **0.8446** | **0.8441** |  | **0.8561** | **0.1859** | **0.8114** | **0.8104** |  | **0.8264** | **0.4995** | **0.7745** | **0.7721** |
|  | Misclassification | **0.0071** | **0.0232** | **0.007** | **0.007** |  | **0.013** | **0.0309** | **0.0137** | **0.0137** |  | **0.0105** | **0.0225** | **0.0113** | **0.0112** |  | **0.0161** | **0.028** | **0.0186** | **0.0186** |
| 2 | Sensitivity | **0.8765** | **0.4466** | **0.8547** | **0.8544** |  | 0.8252 | 0.7041 | 0.798 | 0.7974 |  | 0.8363 | 0.4941 | 0.7963 | 0.7947 |  | 0.8023 | 0.722 | 0.7528 | 0.7509 |
|  | Specificity | **0.9941** | **0.999** | **0.9941** | **0.9941** |  | 0.9933 | 0.9967 | 0.9933 | 0.9934 |  | 0.9915 | 0.9982 | 0.9919 | 0.9921 |  | 0.9915 | 0.9951 | 0.9919 | 0.992 |
|  | PPV | **0.9418** | **0.9802** | **0.9455** | **0.9454** |  | 0.9265 | 0.9569 | 0.9276 | 0.9282 |  | 0.9226 | 0.9653 | 0.9317 | 0.9327 |  | 0.9102 | 0.9407 | 0.9125 | 0.9136 |
|  | YDI | **0.8706** | **0.4456** | **0.8488** | **0.8485** |  | 0.8185 | 0.7007 | 0.7914 | 0.7908 |  | 0.8278 | 0.4923 | 0.7882 | 0.7868 |  | 0.7938 | 0.7171 | 0.7446 | 0.7429 |
|  | Misclassification | **0.0103** | **0.0215** | **0.0111** | **0.011** |  | 0.0175 | 0.0223 | 0.0193 | 0.0193 |  | 0.0142 | 0.0205 | 0.0153 | 0.0152 |  | 0.0208 | 0.0225 | 0.0236 | 0.0236 |
| 4 | Sensitivity | 0.7906 | 0.5792 | 0.777 | 0.7755 |  | 0.6659 | 0.6114 | 0.6406 | 0.6399 |  | 0.7693 | 0.6146 | 0.7537 | 0.7448 |  | 0.6639 | 0.616 | 0.6389 | 0.6291 |
|  | Specificity | 0.9851 | 0.9935 | 0.9844 | 0.9845 |  | 0.9888 | 0.9922 | 0.9891 | 0.9891 |  | 0.9823 | 0.9913 | 0.9819 | 0.9821 |  | 0.9869 | 0.9907 | 0.9875 | 0.9876 |
|  | PPV | 0.913 | 0.9386 | 0.9133 | 0.9132 |  | 0.921 | 0.9361 | 0.9211 | 0.9211 |  | 0.8946 | 0.9198 | 0.8976 | 0.8967 |  | 0.9098 | 0.9255 | 0.9113 | 0.9104 |
|  | YDI | 0.7757 | 0.5728 | 0.7614 | 0.76 |  | 0.6548 | 0.6035 | 0.6297 | 0.629 |  | 0.7516 | 0.6059 | 0.7357 | 0.7269 |  | 0.6508 | 0.6067 | 0.6264 | 0.6166 |
|  | Misclassification | 0.0352 | 0.0498 | 0.0373 | 0.0373 |  | 0.057 | 0.0619 | 0.0604 | 0.0605 |  | 0.04 | 0.0481 | 0.0419 | 0.0427 |  | 0.059 | 0.0625 | 0.062 | 0.0633 |
| 8 | Sensitivity | 0.7567 | 0.5834 | 0.7499 | 0.7469 |  | 0.5595 | 0.5019 | 0.5478 | 0.5447 |  | 0.7592 | 0.6248 | 0.748 | 0.7425 |  | 0.5945 | 0.5391 | 0.5713 | 0.5644 |
|  | Specificity | 0.9761 | 0.9856 | 0.9747 | 0.9757 |  | 0.9858 | 0.9893 | 0.9858 | 0.986 |  | 0.9717 | 0.9816 | 0.9703 | 0.9711 |  | 0.9821 | 0.9866 | 0.9829 | 0.9831 |
|  | PPV | 0.9022 | 0.9224 | 0.9008 | 0.9021 |  | 0.9262 | 0.9371 | 0.926 | 0.926 |  | 0.8873 | 0.9067 | 0.887 | 0.8877 |  | 0.9149 | 0.9273 | 0.9152 | 0.9154 |
|  | YDI | 0.7327 | 0.569 | 0.7246 | 0.7226 |  | 0.5453 | 0.4912 | 0.5336 | 0.5306 |  | 0.731 | 0.6064 | 0.7184 | 0.7136 |  | 0.5767 | 0.5257 | 0.5542 | 0.5476 |
|  | Misclassification | 0.0627 | 0.0856 | 0.0651 | 0.0648 |  | 0.1091 | 0.1192 | 0.1117 | 0.1123 |  | 0.0658 | 0.0815 | 0.069 | 0.0694 |  | 0.1042 | 0.1131 | 0.1087 | 0.1101 |
| 16 | Sensitivity | 0.7003 | 0.5971 | 0.7037 | 0.7014 |  | 0.4232 | 0.3853 | 0.4195 | 0.4158 |  | 0.7263 | 0.6364 | 0.7157 | 0.7108 |  | 0.4878 | 0.4432 | 0.4661 | 0.4594 |
|  | Specificity | 0.9746 | 0.9802 | 0.9713 | 0.9738 |  | 0.9872 | 0.9891 | 0.9871 | 0.9873 |  | 0.9701 | 0.976 | 0.9668 | 0.9696 |  | 0.9836 | 0.9863 | 0.9838 | 0.9844 |
|  | PPV | 0.9247 | 0.9325 | 0.9209 | 0.9243 |  | 0.9418 | 0.9461 | 0.9419 | 0.9417 |  | 0.9148 | 0.922 | 0.9099 | 0.9135 |  | 0.9369 | 0.9424 | 0.9363 | 0.9366 |
|  | YDI | 0.675 | 0.5773 | 0.675 | 0.6752 |  | 0.4104 | 0.3744 | 0.4066 | 0.4031 |  | 0.6964 | 0.6123 | 0.6825 | 0.6804 |  | 0.4714 | 0.4295 | 0.4499 | 0.4438 |
|  | Misclassification | 0.0995 | 0.1234 | 0.1011 | 0.0999 |  | 0.1975 | 0.2086 | 0.1988 | 0.1998 |  | 0.0958 | 0.1159 | 0.1011 | 0.1004 |  | 0.1788 | 0.1915 | 0.1857 | 0.1875 |

Note: The scenarios with large differences between the MCHS-P and the MCS-P are boldfaced.

Table 2 Average values of classic performance characteristics over replicas from the MCHS-P, MCS-P, 50% MWS, and Gini coefficient in scenarios with a single cluster

|  |  | Cluster location | Rural | | | |  | Mixed | | | |  | Urban | | | |
| --- | --- | --- | --- | --- | --- | --- | --- | --- | --- | --- | --- | --- | --- | --- | --- | --- |
| Cases | Size | Measures | MCHS-P | MCS-P | Default | Gini |  | MCHS-P | MCS-P | Default | Gini |  | MCHS-P | MCS-P | Default | Gini |
| 600 | 1 | Sensitivity | 0.9981 | 0.9981 | 0.9981 | 0.9981 |  | 0.9524 | 0.9523 | 0.9355 | 0.9355 |  | 0.9241 | 0.9241 | 0.9207 | 0.9207 |
|  |  | Specificity | 0.9974 | 0.9998 | 0.9979 | 0.9981 |  | 0.9927 | 0.9928 | 0.9929 | 0.9929 |  | 0.9931 | 0.9931 | 0.9932 | 0.9932 |
|  |  | PPV | 0.9307 | 0.9831 | 0.9638 | 0.965 |  | 0.8545 | 0.855 | 0.8575 | 0.8575 |  | 0.9262 | 0.9266 | 0.9322 | 0.9319 |
|  |  | YDI | 0.9955 | 0.9979 | 0.996 | 0.9962 |  | 0.9451 | 0.9451 | 0.9284 | 0.9284 |  | 0.9172 | 0.9172 | 0.9139 | 0.9139 |
|  |  | Misclassification | 0.0026 | 0.0002 | 0.0021 | 0.0019 |  | 0.0083 | 0.0082 | 0.0085 | 0.0085 |  | 0.0088 | 0.0087 | 0.0087 | 0.0087 |
|  | 2 | Sensitivity | 0.9675 | 0.9675 | 0.964 | 0.9641 |  | 0.9286 | 0.9286 | 0.9167 | 0.9167 |  | 0.8948 | 0.8945 | 0.8785 | 0.8785 |
|  |  | Specificity | 0.9977 | 0.9997 | 0.9982 | 0.9983 |  | 0.9934 | 0.9934 | 0.9935 | 0.9935 |  | 0.9900 | 0.9901 | 0.99 | 0.9901 |
|  |  | PPV | 0.9331 | 0.966 | 0.9611 | 0.9619 |  | 0.881 | 0.8812 | 0.8851 | 0.8853 |  | 0.9166 | 0.9173 | 0.9198 | 0.9198 |
|  |  | YDI | 0.9652 | 0.9672 | 0.9622 | 0.9624 |  | 0.922 | 0.922 | 0.9102 | 0.9102 |  | 0.8848 | 0.8846 | 0.8686 | 0.8686 |
|  |  | Misclassification | 0.0023 | 0.0003 | 0.0018 | 0.0017 |  | 0.0084 | 0.0084 | 0.0086 | 0.0086 |  | 0.0135 | 0.0134 | 0.014 | 0.0139 |
|  | 4 | Sensitivity | 0.9519 | 0.9519 | 0.9400 | 0.9400 |  | 0.8945 | 0.8944 | 0.8862 | 0.8857 |  | 0.8494 | 0.8494 | 0.8436 | 0.8431 |
|  |  | Specificity | 0.9975 | 0.9984 | 0.9978 | 0.9978 |  | 0.9914 | 0.9915 | 0.9914 | 0.9914 |  | 0.9757 | 0.9756 | 0.9757 | 0.9757 |
|  |  | PPV | 0.8831 | 0.8904 | 0.8983 | 0.8985 |  | 0.8689 | 0.8692 | 0.8705 | 0.8697 |  | 0.8852 | 0.8849 | 0.8883 | 0.8874 |
|  |  | YDI | 0.9494 | 0.9503 | 0.9378 | 0.9378 |  | 0.8859 | 0.8858 | 0.8776 | 0.8771 |  | 0.8251 | 0.8251 | 0.8194 | 0.8188 |
|  |  | Misclassification | 0.0027 | 0.0018 | 0.0025 | 0.0024 |  | 0.0122 | 0.0122 | 0.0125 | 0.0126 |  | 0.0370 | 0.037 | 0.0375 | 0.0376 |
|  | 8 | Sensitivity | 0.9004 | 0.9004 | 0.8904 | 0.8904 |  | 0.8645 | 0.8656 | 0.8634 | 0.8629 |  | 0.8442 | 0.8447 | 0.8428 | 0.8424 |
|  |  | Specificity | 0.9974 | 0.9979 | 0.9977 | 0.9977 |  | 0.9916 | 0.9916 | 0.9914 | 0.9914 |  | 0.9648 | 0.9648 | 0.9645 | 0.9646 |
|  |  | PPV | 0.8801 | 0.8838 | 0.8933 | 0.8935 |  | 0.8912 | 0.8912 | 0.8923 | 0.8915 |  | 0.8758 | 0.8758 | 0.8768 | 0.8768 |
|  |  | YDI | 0.8978 | 0.8983 | 0.8881 | 0.8881 |  | 0.8561 | 0.8572 | 0.8549 | 0.8543 |  | 0.8090 | 0.8094 | 0.8073 | 0.807 |
|  |  | Misclassification | 0.0032 | 0.0028 | 0.003 | 0.003 |  | 0.0142 | 0.0142 | 0.0144 | 0.0145 |  | 0.0557 | 0.0556 | 0.0562 | 0.0562 |
|  | 16 | Sensitivity | 0.8470 | 0.8470 | 0.8443 | 0.8439 |  | 0.8441 | 0.8467 | 0.8529 | 0.8521 |  | 0.8341 | 0.8349 | 0.8389 | 0.8386 |
|  |  | Specificity | 0.9969 | 0.9972 | 0.9972 | 0.9972 |  | 0.991 | 0.991 | 0.9908 | 0.9908 |  | 0.9566 | 0.9566 | 0.9562 | 0.9563 |
|  |  | PPV | 0.8872 | 0.8889 | 0.8961 | 0.8960 |  | 0.9025 | 0.9026 | 0.9048 | 0.9039 |  | 0.8960 | 0.8960 | 0.8964 | 0.8965 |
|  |  | YDI | 0.8440 | 0.8442 | 0.8415 | 0.8411 |  | 0.8351 | 0.8377 | 0.8438 | 0.8429 |  | 0.7907 | 0.7915 | 0.7951 | 0.7948 |
|  |  | Misclassification | 0.0049 | 0.0046 | 0.0047 | 0.0047 |  | 0.0174 | 0.0173 | 0.017 | 0.0171 |  | 0.0750 | 0.0748 | 0.0741 | 0.0741 |
|  |  | Cluster location | Rural | | | |  | Mixed | | | |  | Urban | | | |
| Cases | Size | Measures | MCHS-P | MCS-P | Default | Gini |  | MCHS-P | MCS-P | Default | Gini |  | MCHS-P | MCS-P | Default | Gini |
| 6000 | 1 | Sensitivity | 0.9897 | 0.9897 | 0.9897 | 0.9897 |  | 0.9036 | 0.9036 | 0.8691 | 0.8691 |  | 0.8645 | 0.8644 | 0.8186 | 0.8186 |
|  |  | Specificity | 0.9964 | 0.9998 | 0.9974 | 0.9975 |  | 0.9918 | 0.9919 | 0.9924 | 0.9924 |  | 0.992 | 0.9921 | 0.9925 | 0.9925 |
|  |  | PPV | 0.9217 | 0.9824 | 0.9657 | 0.9676 |  | 0.832 | 0.8327 | 0.8386 | 0.8388 |  | 0.9097 | 0.91 | 0.9205 | 0.9203 |
|  |  | YDI | 0.9861 | 0.9895 | 0.9871 | 0.9872 |  | 0.8954 | 0.8955 | 0.8615 | 0.8615 |  | 0.8565 | 0.8565 | 0.8111 | 0.8111 |
|  |  | Misclassification | 0.0036 | 0.0004 | 0.0026 | 0.0025 |  | 0.0103 | 0.0102 | 0.0106 | 0.0105 |  | 0.0114 | 0.0113 | 0.0121 | 0.0121 |
|  | 2 | Sensitivity | 0.9434 | 0.9434 | 0.9252 | 0.9252 |  | 0.8876 | 0.8873 | 0.8557 | 0.8556 |  | 0.8479 | 0.8475 | 0.8062 | 0.8062 |
|  |  | Specificity | 0.9968 | 0.9992 | 0.9977 | 0.9977 |  | 0.9926 | 0.9927 | 0.9929 | 0.993 |  | 0.9903 | 0.9904 | 0.9908 | 0.9908 |
|  |  | PPV | 0.9072 | 0.9392 | 0.9449 | 0.9461 |  | 0.8585 | 0.8588 | 0.8662 | 0.8665 |  | 0.9055 | 0.9058 | 0.913 | 0.9129 |
|  |  | YDI | 0.9402 | 0.9426 | 0.9228 | 0.9229 |  | 0.8802 | 0.88 | 0.8487 | 0.8487 |  | 0.8382 | 0.8379 | 0.797 | 0.797 |
|  |  | Misclassification | 0.0032 | 0.0008 | 0.0024 | 0.0023 |  | 0.0103 | 0.0102 | 0.0108 | 0.0108 |  | 0.0149 | 0.0148 | 0.0159 | 0.0159 |
|  | 4 | Sensitivity | 0.9126 | 0.9125 | 0.8747 | 0.8748 |  | 0.854 | 0.8542 | 0.8286 | 0.8284 |  | 0.8161 | 0.8159 | 0.8017 | 0.8012 |
|  |  | Specificity | 0.9968 | 0.9977 | 0.9975 | 0.9975 |  | 0.9904 | 0.9905 | 0.9909 | 0.9909 |  | 0.9765 | 0.9766 | 0.9769 | 0.9768 |
|  |  | PPV | 0.8484 | 0.8543 | 0.8702 | 0.8708 |  | 0.8497 | 0.8503 | 0.8561 | 0.8561 |  | 0.8778 | 0.8778 | 0.883 | 0.882 |
|  |  | YDI | 0.9094 | 0.9102 | 0.8722 | 0.8723 |  | 0.8443 | 0.8446 | 0.8195 | 0.8193 |  | 0.7926 | 0.7924 | 0.7786 | 0.7781 |
|  |  | Misclassification | 0.0036 | 0.0027 | 0.0031 | 0.003 |  | 0.0148 | 0.0147 | 0.0152 | 0.0152 |  | 0.0396 | 0.0395 | 0.0406 | 0.0407 |
|  | 8 | Sensitivity | 0.8706 | 0.8705 | 0.838 | 0.838 |  | 0.8406 | 0.8411 | 0.8253 | 0.8252 |  | 0.8239 | 0.824 | 0.8152 | 0.8151 |
|  |  | Specificity | 0.9965 | 0.9971 | 0.9971 | 0.9971 |  | 0.9907 | 0.9907 | 0.9908 | 0.9908 |  | 0.9623 | 0.9624 | 0.9625 | 0.9626 |
|  |  | PPV | 0.8524 | 0.857 | 0.8695 | 0.8707 |  | 0.8775 | 0.8773 | 0.881 | 0.8807 |  | 0.865 | 0.8647 | 0.8673 | 0.8673 |
|  |  | YDI | 0.867 | 0.8676 | 0.835 | 0.8351 |  | 0.8314 | 0.8319 | 0.8162 | 0.816 |  | 0.7863 | 0.7863 | 0.7777 | 0.7777 |
|  |  | Misclassification | 0.0044 | 0.0038 | 0.0041 | 0.004 |  | 0.0161 | 0.0161 | 0.0167 | 0.0167 |  | 0.0612 | 0.0612 | 0.0625 | 0.0625 |
|  | 16 | Sensitivity | 0.8306 | 0.8307 | 0.8084 | 0.8083 |  | 0.8251 | 0.8263 | 0.8179 | 0.8179 |  | 0.8264 | 0.827 | 0.8226 | 0.8224 |
|  |  | Specificity | 0.9959 | 0.9961 | 0.9964 | 0.9965 |  | 0.9902 | 0.9902 | 0.9903 | 0.9903 |  | 0.9571 | 0.957 | 0.9569 | 0.957 |
|  |  | PPV | 0.8579 | 0.8595 | 0.8709 | 0.8716 |  | 0.8907 | 0.8907 | 0.894 | 0.8938 |  | 0.8954 | 0.8951 | 0.8957 | 0.8958 |
|  |  | YDI | 0.8265 | 0.8268 | 0.8048 | 0.8048 |  | 0.8153 | 0.8166 | 0.8082 | 0.8082 |  | 0.7835 | 0.7841 | 0.7794 | 0.7794 |
|  |  | Misclassification | 0.0061 | 0.0059 | 0.0059 | 0.0058 |  | 0.0192 | 0.0191 | 0.0196 | 0.0196 |  | 0.0767 | 0.0765 | 0.0778 | 0.0778 |

Table 3 Average performance measures for each MWS in 600-two-1

| size | MCHS-P | MCS-P | Sensitivity | Specificity | PPV | YDI | Misclassification |
| --- | --- | --- | --- | --- | --- | --- | --- |
| 1 | 0.4535 | 0.6151 | 0.0034 | **0.9999** | 0.9738 | 0.0033 | 0.0267 |
| 2 | 0.4534 | **0.6153** | 0.0034 | 0.9998 | 0.9762 | 0.0032 | 0.0268 |
| 3 | 0.6094 | 0.4242 | 0.8904 | 0.9998 | **0.9938** | 0.8902 | **0.0031** |
| 4-12 | 0.6106-0.6117 | 0.4186-0.4226 | 0.8944-0.902 | 0.9971-0.9992 | 0.9527-0.9773 | 0.8935-0.8991 | 0.0036-0.0055 |
| 13 | **0.6118** | 0.4186 | 0.902 | 0.997 | 0.9526 | 0.8991 | 0.0055 |
| 14 | **0.6118** | 0.4185 | 0.9023 | 0.9969 | 0.9518 | 0.8992 | 0.0057 |
| 15 | **0.6118** | 0.4184 | 0.9025 | 0.9967 | 0.9512 | **0.8993** | 0.0058 |
| 16 | **0.6118** | 0.4184 | **0.9026** | 0.9967 | 0.951 | **0.8993** | 0.0058 |
| 17 | 0.6115 | 0.4192 | 0.9003 | 0.9967 | 0.9519 | 0.897 | 0.0059 |
| 18-24 | 0.6115 | 0.4191-0.4193 | 0.9003-0.9007 | 0.9963-0.9966 | 0.9508-0.9516 | 0.8969-0.8971 | 0.006-0.0063 |
| 25-50 | 0.6116 | 0.4187-0.4191 | 0.9007-0.9015 | 0.9955-9962 | 0.9491-0.9507 | 0.897-0.8972 | 0.0063-0.007 |

Note: For each performance, the optimal value is shown in bold. This table shows that in a scenario with highly heterogeneous clusters, the MCHS-P selected the best MWS, whereas the MCS-P selected the nearly worst MWS.

**Supplementary file information**

- Supplementary file 1: The simulation datasets for the complex scenario [XLSX].
- Supplementary file 2: The average values of classic performance characteristics over replicas from the MCHS-P, MCS-P, 50% MWS, and Gini coefficient in Kulldorff’s benchmark datasets and the average performance measures for each MWS in 600-two-1[PDF].
- Supplementary file 3: The result of simulation study: the average values of the MCHS-P, MCS-P and five classic performance measures over replicas under each selected MWS for each Kulldorff’s simulation scenarios [XLSX].
- Supplementary file 4: The datasets and result of the additional simulation scenario, 6000-three-16RR3.0-2.0-1.2: the simulation dataset and the average values of the MCHS-P, MCS-P and five classic performance measures over replicas under each selected MWS [XLSX].
